# Selected Aspects of Chemoresistance Mechanisms in Colorectal Carcinoma—A Focus on Epithelial-to-Mesenchymal Transition, Autophagy, and Apoptosis

**DOI:** 10.3390/cells8030234

**Published:** 2019-03-12

**Authors:** Veronika Skarkova, Vera Kralova, Barbora Vitovcova, Emil Rudolf

**Affiliations:** Department of Medical Biology and Genetics, Charles University, Faculty of Medicine in Hradec Králové, Zborovská 2089, 500 03 Hradec Králové, Czech Republic; hanusovav@lfhk.cuni.cz (V.S.); kralovav@lfhk.cuni.cz (V.K.); vitovcob@lfhk.cuni.cz (B.V.)

**Keywords:** colorectal carcinoma (CRC), chemoresistance, epithelial-to-mesenchymal transition (EMT), autophagy, apoptosis

## Abstract

Chemoresistance has been found in all malignant tumors including colorectal carcinoma (CRC). Nowadays chemoresistance is understood as a major reason for therapy failure, with consequent tumor growth and spreading leading ultimately to the patient’s premature death. The chemotherapy-related resistance of malignant colonocytes may be manifested in diverse mechanisms that may exist both prior to the onset of the therapy or after it. The ultimate function of this chemoresistance is to ensure the survival of malignant cells through continuing adaptation within an organism, therefore, the nature and spectrum of cell-survival strategies in CRC represent a highly significant target of scientific inquiry. Among these survival strategies employed by CRC cells, three unique but significantly linked phenomena stand out—epithelial-to-mesenchymal transition (EMT), autophagy, and cell death. In this mini-review, current knowledge concerning all three mechanisms including their emergence, timeline, regulation, and mutual relationships will be presented and discussed.

## 1. Introduction

Colorectal carcinoma (CRC) is a malignant neoplasm originating from colonic mucosal epithelia via dysregulated colonocyte proliferation, differentiation, and migration. At present, CRC represents a considerable health burden in many developed as well as a number of developing countries, where it ranks among the top causes of premature morbidity and mortality [1,2]. Most newly diagnosed CRC cases are classified as sporadic [3], with no discernible and identified individual predispositions, while in the remaining cases family history and/or hereditary patterns are featured [4,5]. The development of a sporadic form of CRC ranges from years to decades, and is believed to involve sequential changes both in cell autonomous mechanisms as well as microenvironmental signals. At the cellular level, the CRC malignant process begins with altered proliferation and loss differentiation of colonocytes, which gradually accumulate forming abnormal clusters of cells within the crypt epithelium (i.e., aberrant crypt foci). Over time, selected foci may grow through expansive enlargement, thus forming benign tissue lesions—hyperplastic polyps or adenomatous polyps. To advance further, cells in a particular polyp have to undergo various stages of dysplasia to drive the transformation of benign polyps first into adenomas, then adenocarcinomas, and finally invasive carcinomas [6,7,8]. Most CRCs develop from adenomatous polyps, although an alternative pathway including hyperplastic polyps and serrated adenomas has been described [9]. Each histopathological stage of CRC development is accompanied or underpinned by cell-specific molecular changes in concrete genes and signaling pathways, including various mutations and/or epigenetic alterations. Detailed studies have recently led to definition of the three main genetic pathways leading to the development of CRC: the chromosomal instability pathway (CIN), serrated pathway (CPG island methylator phenotype pathway—CIMP), and microsatellite instability pathway (MSI). While each of these pathways accounts for a certain proportion of identified sporadic CRC cases, the CIN pathway seems to prevail, being present in up to 90% of all reported cases [10]. The development of the CIN phenotype in colonocytes relies upon a series of genetic and cellular mechanisms including unrepaired DNA damage and/or the defective behavior of chromosomes, both mechanisms that enable the transition of normal mucosal colonocytes first into aberrant hyperplastic and then to dysplastic cells. This transition often occurs via a mutant adenomatous polyposis coli gene (*APC*) whose non-functional product cannot participate in the protein complex-mediated sequestration and degradation of cytosolic β-catenin. The resulting increased finally, inhibited exfoliation. The progress of polyps to the adenoma stage requires additional transcriptional activity of β-catenin leads to a prolonged activation of the wingless/integrated signaling pathway (Wnt) [11], endowing the cells with a stem cell-like phenotype. This stimulates further changes in the cells’ biological behavior, namely arrested differentiation, suppressed migration, and, mutations in cyclooxygenase-2 (*COX-2*—early stage), v-raf murine sarcoma viral oncogene homolog B1/ v-Ki-ras2 Kirsten rat sarcoma viral oncogene homolog (*BRAF/K-RAS*—intermediate stage), as well as the cell division control protein 4/sma- and mad-related protein 4 (*CDC4/SMAD4*—late stage) genes. Several other events contribute, i.e., changes in the activity of transforming growth factor beta receptor 2 (TGFBR2), along with the inactivation and hypermethylation of mismatch repair genes, often in the context of microenvironmental tissue remodeling driven for instance by inflammation and modified by microbiome [12,13,14,15,16]. The final carcinoma stage is then marked by the constitutive activation of phosphatidylinositol-4,5-bisphosphate 3-kinase/ phosphatase and tensin homolog (PI3K/PTEN) pathway in the cells and their uncontrolled cell cycle progress due to mutations in tumor protein 53 (*TP53*) or other defects in TP53-dependent signaling [17]. The stated sequence of molecular and cellular events leading target colon mucosa cells from the normal to full malignant phenotype is not meant to be an exhaustive and in-depth analysis of all the involved molecules and mechanisms, which have been summarized and discussed elsewhere [3,18,19,20]. The main purpose is to introduce the basic biology of CRC and call attention to the fact that the mentioned CRC genesis may not always occur in such a linear manner; this irregularity can be attributed to the considerable heterogeneity of the malignant process itself, which entails a number of identified (and perhaps some still unidentified) molecules and mechanisms along with microenvironmental influences. All of these factors finally combine in various ways to generate specific selective pressures acting to favor the most advantageous (i.e., adapted) cell clones, a process that further drives CRC progression via the local spread and systemic dissemination throughout the entire organism. Despite the fact that the previously mentioned mutations in certain genes do play specific roles in the development of CRC, their presence and respective roles vary significantly in individual analyzed cases. This is mainly due to the marked intertumor and intratumor genetic heterogeneity of CRC, in which only select driver mutations presenting evolutionary advantages for the malignant process itself are shared by the majority of tumors [21,22]. Accordingly, it has been proposed that an average CRC may harbor up to 80 mutations, with fewer than 15 of them the driving force for tumorigenesis. Moreover, the mutational profile of any two CRC primary tumors shows minimal overlap, and the majority of existing mutations are essentially discrete to one specific tumor [23]. Together with the reprogrammed biology concerning proliferation, differentiation, migration, and ultimately invasion, malignant colonocytes show increased aggressiveness, namely an enhanced resistance to various noxious signals, a process which favors their survival and spread. In the following parts of this mini-review, selected mechanisms of this chemoresistance, in particular those associated with the currently available knowledge, will be presented and discussed.

## 2. Chemoresistance of CRC Cells

Chemotherapy continues to be one of the mainstays in the treatment of the majority of solid tumors including CRC. CRC-specific chemotherapeutical regimens in the form of adjuvant therapy are administered to patients with stage III and IV disease; these include the use of individual classic cytotoxic agents alone or in combination. Among these drugs are antimetabolites 5-fluorouracil (5-FU) irreversibly inhibiting thymidylate synthase (TYMS), its prodrug capecitabine (CA) which presents comparable efficacy but lower toxicity compared to 5-FU [24], and raltitrexed used in case of a lack of tolerance to the previous agents [25]. 5-FU is also combined with 5-formyltetrahydrofolate (folinic acid or Leucovorin (LEU)), which was found to improve response rate and the overall survival of patients compared to 5-FU alone [26]. Further improvements in the overall survival of stage III CRC patients was achieved by addition of oxaliplatin (OX), an alkylating agent inducing DNA damage in exposed cells [27]. Conversely, the semisynthetic analog of camptothecin irinotecan (IRI), which inhibits topoisomerase I, thereby blocking DNA repair and replication in treated cells, did not seemingly produce any major benefit to patients’ survival [28]. These enumerated individual agents are nowadays most often employed in the following combined regimens: FOLFOX (5-FU/LEU/OX), FOLFIRI (5-FU/LEU/IRI), FOLFIRINOX (5-FU/LEU/OX/IRI), or XELOX (OX/CA) [25]. In addition, patients with advanced or metastatic CRC are often treated with newly developed targeted biologicals which include recombinant monoclonal antibodies, small molecule inhibitors and immuno therapeutics [29]. In addition, among these treatments, bevacizumab inhibits the vascular endothelial growth factor (VEGF) and cetuximab or panitumumab inhibit the epidermal growth factor receptor (EGFR). These agents are used either alone or in combination with standard chemotherapy, with the best results obtained in select patient groups positive for *KRAS/NRAS* and *BRAF* mutations [25]. Alternatively, since 2013 the multi tyrosine kinase inhibitors regorafenib and aflibercept are available for treatment of patients with refractory metastatic CRC [30]. Moreover, quite recently (2017) pembrolizumab, a humanized antibody targeting programmed the cell death 1 (PD-1) receptor of lymphocytes, was approved for unresectable or metastatic CRC with mismatch repair deficiency or microsatellite instability [31]. A detailed description of the currently used compounds and their mechanisms of action along with their actual applications in various treatment protocols was not a subject of the present review; an interested reader is thus referred to relevant published summaries for further information on this subject [32,33].

Irrespective of the number and the mechanism of the employed drugs or their combinations, the basic and ultimate goal of all chemotherapy is simple—to inhibit the aberrant proliferation and spread of malignant cells throughout the body. In the best case it is hoped that employed drugs (in addition to other established approaches such as surgery and radiotherapy) will not only permanently stop cancer growth, reproduction, and other activities including the metastasis of malignant cells, but will remove those cells altogether from the treated human body. While this concept appears technically amenable due to a number of specific changes in malignant cells that often make them a relatively distinct and easy target for chemotherapy, in reality an effective treatment of many malignancies including CRC is hampered by the presence of chemoresistance.

At present, the chemoresistance of malignant cells is recognized as one of the most important reasons for chemotherapeutic failure and consequent disease progression followed by the untimely death of a patient [34]. Found in all malignant tumors including CRC, chemoresistance is understood as a series of existing or newly developed features and behavioral patterns of malignant cells that ensure their increased survival in the “hostile” environment of the host organism [35,36]. Furthermore, ample evidence exists that, apart from malignant cells themselves, a number of tumor cell-independent factors could influence or directly cause this chemoresistance via various mechanisms. These include but are not limited to several microenvironment-originating players, such as signals from stromal cancer-associated fibroblasts (CAFs), adipocytes, and various modified white blood cells, as well as defective vasculature with resulting hypoxia and inflammation [37,38,39]. Traditionally, chemoresistance is classified as either an intrinsic phenomenon (i.e., therapy-independent) or acquired one (i.e., chemotherapy-related or dependent) in both cell autonomous as well as independent variants [40,41,42].

**Figure 1:**
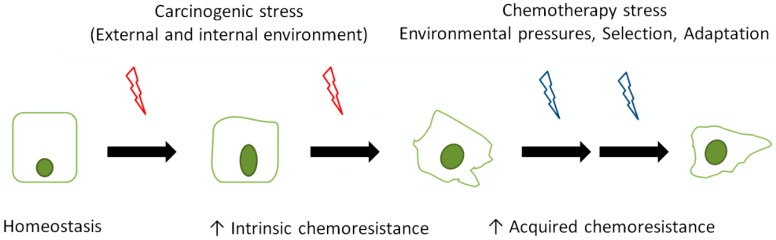
Development of intrinsic and acquired chemoresistance in the process of colorectal carcinogenesis. During malignant conversion, individual cells are exposed to carcinogenic events from external and internal environments, which prompt the gradual development of intrinsic chemoresistance to mediate cell survival. During advanced stages of carcinogenesis and upon exposure to chemotherapy-related stress combined with selection-adaptation-related pressures, malignant cells develop acquired chemoresistance.

The intrinsic chemoresistance of CRC develops over the time and probably closely follows the individual stages of the malignant process. It is thus reasonable to assume that CRC cells in more advanced stages would show more extensive resistance, due to the considerable genotypic and phenotypic heterogeneity in individual tumors, however, the timing and staging of intrinsic resistance development is very difficult to map since it encompasses a range of the aforementioned cellular features as well as particular environmental influences (Figure 1). Thus, owing to serial genetic and epigenetic alterations that underlie the reprogramming of the colonocytes under transformation, CRC cells exhibit an increased resistance against external inhibitory signals (including cytotoxic drugs) via diverse mechanisms, many of which are related directly to the used individual cytostatics or targeted agents. Thus, resistance to F-5U, OXA, or IRI may occur due to enhanced cellular efflux (see below), as well as the intracellular metabolism, upregulation, or alteration of their intracellular targets, increased dihydropyrimidin dehydrogenase and thymidylate synthase activities, increased levels of reduced glutathione, or increased nucleotide excision repair [43]. The methylation-driven inactivation of the gene encoding thymidine phosphorylase, which is responsible for the activation of capecitabine, causes the resistance of chemotherapy-naïve CRC cells to this drug [44]. In case of the monoclonal antibodies cetuximab, panitumumab, and bevacizumab, a number of resistance mechanisms have been reported, including mutations in *KRAS*, *BRAF*, *EGFR* genes, loss of *PTEN*, activation of *IGF1R*, amplification of *MET*, alteration of *VEGF/VEGFR*, as well as changes in the respective signaling pathways [45]. The presence of inherent chemoresistance to the kinase inhibitors (aflibercept and regorafenib) in CRC cells is supposed to occur via several of the previously mentioned mechanisms, despite the fact that the exact nature of the chemoresistance is difficult to assess given the number of blocked targets and their potential crosstalk [43]. Another aspect of the inherent chemoresistance of CRC is the presence of stem cell-like cancer cells in a particular tumor mass, which represent a heterogeneous population of cells with overlapping and sometimes unique combinations of markers. These cells are known to be resistant to chemotherapy owing to the high expression of drug efflux and antiapoptotic proteins (see below), aldehyde dehydrogenase activity, enhanced DNA repair, and the activation of several pro-survival signaling molecules such as nuclear factor kappa B (NF-κB). Additionally, the cells may rapidly enter a state of quiescence that makes them unresponsive to rapidly dividing cell therapies [46]. Finally, other general events such as dysregulated death signaling, enhanced survival activities (i.e., autophagy, proliferative wiring), and/or phenotypic plasticity have been reported in CRC cells; these events complement the above mentioned intrinsic mechanisms of chemoresistance [6,47,48,49,50].

Acquired chemoresistance in tumor cells develops as a direct consequence of their exposure to chemotherapy and as such manifests in a number of specific as well as nonspecific ways that in the pertinent literature have often been linked to the pharmacokinetic or pharmacodynamic profiles of a particular drug. In addition to the above mentioned concrete examples, the most often cited mechanisms are the expression and activity of ATP-binding cassette (ABC) transmembrane proteins (i.e., P-glycoprotein) and organic cation transporters (i.e., 1,2,3, copper efflux transporters, P-type ATPases such as ATP7A and ATP7B, as well as the multidrug resistance-associated protein (MRP)), all of which actively remove the drugs from the cell [51,52]. In addition, due to chemotherapy-related mutagenesis in exposed cancer cells and their adaptive responses, such as the activation of compensatory signaling pathways, and extensive alterations of drug intracellular targets, increased tolerance to drug-inflicted damage as well as lower drug access to the cells were reported in the treated CRC cells, and were thus responsible for the present chemoresistance [35]. Recently, prognostic differences and related chemoresistance to anti-EGFR therapies in sidedness have been identified, with a worse prognosis and less responsiveness for malignant tumors on the right side of the colon [53]. It has been proposed that until several potential biological variables potentially responsible for this treatment effect (i.e., *BRAF* and *NRAS* mutations and the CpG island methylator phenotype (CIMP)) are elucidated, patients whose primary cancers arise in the right side of the colon should not be treated with cetuximab or panitumumab in the first-line setting [45].

Since chemotherapy is routinely supplied to cells of advanced (often metastatic) CRC stages during which a mixture of various stated intrinsic and drug-dependent mechanisms likely exists, it is difficult to ascertain their order of importance and assign them particular roles. This is especially true since these mechanisms are constantly shuffled and refined in the face of tumor microevolution to ensure malignant cell survival via continuing adaptation. This is why the nature and spectrum of cell-survival strategies in CRC as well as in other malignancies represent a very prominent target of scientific inquiry. Three unique but significantly linked phenomena have thus become especially important—epithelial-to-mesenchymal transition (EMT), autophagy, and cell death.

## 3. Epithelial-to-Mesenchymal Transition

EMT is a process that drives a cellular trans-differentiation continuum under physiological conditions (embryogenesis—type I, or tissue healing and regeneration, inflammation and fibrosis—type II) and pathological states (cancer invasion and metastasis—type III) [54]. During EMT epithelial cells gradually undergo the loss of their typical morphological features (cell polarity, membrane adhesion, cell-to-cell contacts) and develop a mesenchymal phenotype with the typical cellular stellate morphology, different propensity for intercellular signaling, as well as overall distinct cyto and tissue architecture. In addition, the transformed cells typically display an increased motility, enhanced synthetic activity, matrix remodeling properties, and a propensity for invasiveness [55]. These phenotypic alterations depend on *en masse* changes in the expression of many genes due to the activated genetic as well as epigenetic mechanisms [56]. Many of these concern the specific transcription factors (i.e., Snail, Slug, ZEB1/2, Twist1/2) whose expression and cellular localization drive the resultant down-regulation or loss of epithelial surface markers (E-cadherin, claudins, occludins, and cytokeratins) as well as the up-regulation of mesenchymal features (i.e., N-cadherin, vimentin, fibronectin, smooth muscle actin, and integrins) and extracellular matrix components (i.e., specific collagens) [57,58]. EMT in CRC malignant colonocytes may be induced by different stimuli originating from external sources such as *transforming growth factor β* (TGF-β) as well as various cytokines acting in concert with intracellular operative signaling cascades including phosphatidylinositol-4,5-bisphosphate 3-kinase (PI3K), the nuclear factor kappa-light-chain-enhancer of activated B cells (NF-κB), along with other stimuli [56,59]. Still more importantly, EMT may be induced by individual cytotoxic drugs used in CRC therapy, as demonstrated in the case of chronic OPT exposure, which led to the emergence of cells with the characteristic phenotypic alterations associated with EMT such as loss of polarity and increased mobility as well as decreased E-cadherin and increased Snail and vimentin expressions [60]. Along the same lines, the treatment of colon cancer cells HCT-116 with doxorubicin induced EMT cell phenotypes, TGF-β signaling along with significantly increased multi-drug resistant plasma membrane glycoprotein levels [61]. Moreover, in contrast to parental HT-29 colon adenocarcinoma cells, 5-FU-resistant HT-29 cells showed an increased expression of several mesenchymal markers as well as the EMT-inducing transcription factors Twist, ZEB1, and Zeb2, as well as enhanced migration [62].

Malignant cells with an activated EMT program are not only capable of migration and invasion to the adjacent tissues, but also show elevated chemoresistance. The reported concrete reasons for this nascent ability of cells to withstand induced damage are neither universal nor entirely specified. Proposed mechanisms include the context-dependent stemness of the transformed cells and their mesenchymal status, the dysregulation of particular transcription factors, as well as relevant signaling cascades influencing major antitumor barriers in cells, i.e., senescence and various forms of cell death [63,64,65,66,67]. In CRC, robust evidence exists that both tumor progression and therapeutic resistance associate with EMT [68]. Reduced expression of E-cadherin was shown to be a negative prognostic factor in several studies of colon adenocarcinoma [69,70,71]. Additionally, the relationship between the expression of cadherins and their clinical significance in CRC has also been published, with the altered levels of E- and N-cadherins in malignant tissue correlating significantly with local infiltration depth, tumor stage, vascular invasion, tumor grade, and CA19-9 blood level [72]. Moreover, in CRC patients an increased N-cadherin presence was linked with an advanced stage of TNM, lymph nodes metastasis, and distant metastasis [73]. Conversely, forced Snail expression in malignant colonocytes enhanced OPT resistance, thus demonstrating that EMT mediators are directly involved in the therapeutic resistance of CRC [74]. Thus, the mentioned experimental as well as clinical evidence clearly suggests that the expression of select EMT markers not only associates with the clinical course of CRC in terms of its propensity to invade and metastasize, but is also related to its aggressiveness, i.e., chemoresistance. In this respect it should be emphasized that should the expression of EMT markers such as E-cadherin be used to assess the particular patient risk of metastatic disease and its aggressiveness, a clinically-optimal cut point for such a marker needs to be carefully determined [75].

## 4. Autophagy

Autophagy is a general term encompassing several forms of regulated catabolic processes that ensure the recycling of damaged, aged, malfunctioning or otherwise redundant cytoplasmic materials, molecules, and organelles in eukaryotic cells. In all recognized autophagy forms, a series of steps, including the formation of membrane vesicles and recognition protein complexes, ensures the transport of the target substrates to lysosomes, where they are degraded [76]. On the molecular level, autophagy is regulated by almost 40 autophagy-related genes (ATG), the products of which sense, transduce, and execute individual steps of the autophagy cascade, where the master regulator is a mammalian target of rapamycin (mTOR) and PI3K complex [77]. Low level autophagic recycling occurs in all cells almost constantly, but at times it may be significantly enhanced by various physiological and pathological stimuli with diverse outcomes, both protective as well as destructive [78]. The role of autophagy in the malignant transformation of cells is dichotomous, as at individual stages of cancer development autophagy may play both tumor suppressing and promoting roles [79]. The explanation of this duality is usually based on the stated context-dependent signals, the stage-specific status of individual ATG or related genes controlling autophagy itself, or general cancer development [80,81,82].

In the process of carcinogenesis, autophagy may initially play a tumor suppressing role via its cell quality surveillance mechanisms combating various stresses originating from the exposure of cells to adverse external conditions. These mechanisms range from, at one extreme, the recycling of damaged or dangerous molecules and organelles, a process that sustains positive energy balance and survival, to, at the other, promoting cell senescence and death. Autophagy may also help to reduce invasion and metastasis by promoting inflammatory responses against tumors. Furthermore, autophagy has been known to limit tumor necrosis and the expansion of dormant cancer cells into micrometastases as well as to impair oncogene-induced senescence [83].

At later stages of carcinogenesis, autophagy may act to promote tumor formation by providing energy and nutrients important to the metabolism and growth of malignant cells, or by inhibiting cellular demise and increasing drug resistance [78]. Furthermore, autophagy may support metastasis during advanced stages of cancer by increasing the survival of detached metastatic cells in the absence of an extracellular matrix, for instance by their transition to a dormant or senescent state until appropriate conditions occur [84]. Also, cancer stem-like cells often show an elevated autophagic flux, and their ability to form tumors in vivo appears to be associated with autophagy, as demonstrated by the prevention of tumor formation through the genetic inhibition of *BECN1* or *ATG4A* [85]. Thus, autophagy may also contribute to tumor progression by maintaining the viability of the cancer stem cells. Lastly, the pro-malignant role of autophagy has been verified in experimental studies in which the inhibition of autophagy was linked to reduced tumor processes [86]. Ample evidence exists that autophagy is upregulated in several established colon cancer cell lines representing diverse stages of CRC development, and the pharmacological or genetic suppression of autophagy in vitro has been shown to increase the chemosensitivity of these cells in various experimental settings, including the use of standard cytotoxic agents [87,88,89,90,91]. Moreover, the loss of the tumor suppressor *APC* in mice activates autophagy and promotes the initiation and progression of intestinal cancer. Conversely, *ATG7* deficiency prevents the tumor initiation and progression induced by *APC* loss via the activation of specific anti-tumor T response and microbiota imbalance [92].

The existence of similarly increased autophagy in vivo in human CRC tissues is far more controversial, since due to many technical constraints it is not possible to verify the dynamics of autophagy flux in situ in biopsied tissues; the confirmation of these dynamics has been proposed as the standard proof of the presence of this process in cells [93]. Also, the differences that exist in biopsy sampling processes, the heterogeneity of CRC, along with the particular composition of a tumor mass, which often also contains a considerable portion of non-malignant cells, are all factors that make the identification of autophagy and its rate in human samples complicated. Identification is essentially dependent on the expression analysis of a number of selected biomarkers, including the autophagy relevant microtubule-associated proteins 1A/1B light chain (*MAP1LC3B*), Beclin-1 (*BECN1*), autophagy related 5 (*ATG5*), and B-cell lymphoma 2 (*BCL-2*) genes, as well as others whose expression levels in CRC biopsy sections are used as a surrogate for the active autophagy [94]. Here, the data from clinical as well as in vitro experimental studies are often conflicting. On the one hand, the higher expression patterns of individual analyzed genes correlated in some reports with advanced stages of CRC, chemotherapy with particular drugs, as well as poor prognosis and survival of patients [94,95,96,97]. Conversely, opposite outcomes have also been published [98,99,100]. The same disparity in results has also been documented in cases of the detected low expression of the same autophagy marker molecules [101,102]. Despite the convincing data from in vitro experiments using various colon cancer cell lines and various forms of autophagy induction or inhibition, it appears that the regulation, relevance, and contribution of autophagy to the development of CRC and, in particular, the related chemoresistance remain far from elucidated, with further studies needed to (A) ascertain the presence and regulation of autophagy in all stages of the CRC malignant process, to (B) determine the contribution of this process to native and acquired chemoresistance in malignant colonocytes and, finally, to (C) exploit this process in the treatment of CRC [103,104,105].

## 5. Cell Death

At present, cell death is understood not only as a final point of existence of all types of cells but also as a complex of multiple phenotypical cellular modalities with diverse triggering stimuli, intricate regulation, and mutual relationships. According to recently updated nomenclature regarding cell death, there are twelve recognized programmed and non-programmed cell death modalities (subroutines), each with specified features, signaling, and regulation. Furthermore, a number of other modalities are also acknowledged to exist without a clear understanding of their phenotypic features, regulation, occurrence, and physiological or pathological context [106]. In normal colonic epithelia, apoptosis/anoikis and/or necroptosis are reported to be present, which dispose of old colonocytes or defective cells to enable physiological cell turnover in this tissue [107,108]. All of the mentioned cell death modalities are to date mechanistically and molecularly characterized, and have been reviewed in a number of published papers [109,110,111,112,113,114]. In this respect, the process of malignant transformation of colonic tissues into CRC includes alterations in the expression and/or activity of a number of cell death-related genes, molecules, and pathways leading to dysregulation and decrease in cell death rates. In cases of apoptosis, the most commonly reported alterations concern the *TP53*, bcl-2-like protein 4 (*BAX*) [115,116], *BCL-2* [96] p53 upregulated modulator of apoptosis PUMA (*BB3*) [117], cellular caspase 8 (FLICE)-like inhibitory protein (*CFLAR*) [118], the X-linked inhibitor of the apoptosis protein (*XIAP*) [119], cellular inhibitor of apoptosis 2 (*CIAP2*) [120], the baculoviral inhibitor of apoptosis repeat-containing 5 SURVIVIN (*BIRC5*), and second mitochondrial derived activator of caspases (*SMAC*) genes [121,122]. In addition, marked changes in the expression of the necroptosis specific *RIPK1* and *RIPK3* molecules in CRC tissues have recently been published [109]. Collectively, these reports clearly suggest that the development of CRC is inherently associated with the suppression of cell death in malignant colonocytes, thus constituting one important cause of their primary chemoresistance.

## 6. EMT, Autophagy, and Cell Death in Shaping Chemoresistance

**Figure 2:**
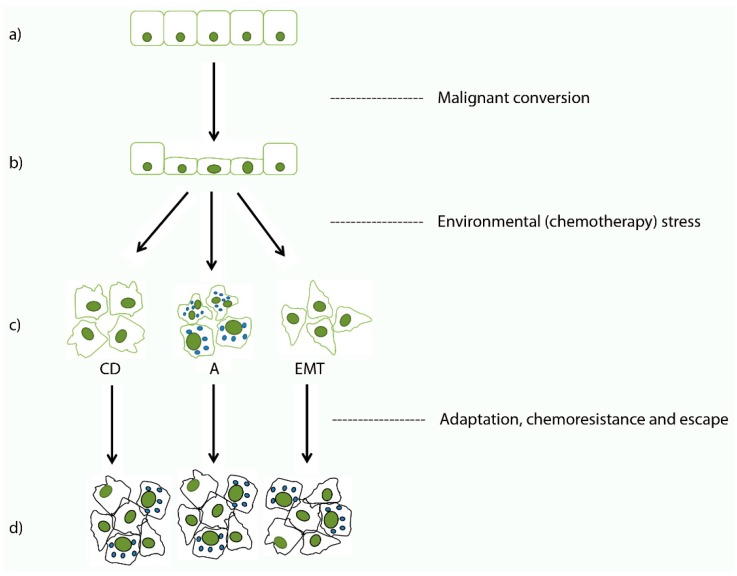
Selected phenotype malignant colorectal carcinoma cells develop to escape chemotherapy-dependent effects. Normal colonic epithelial cells (**a**) undergo malignant conversion (**b**) and upon exposure to various stresses (environment and/or chemotherapy-related) they became resistant to cell death (CD) or upregulate autophagy (A) or undergo epithelial-mesenchymal transition (EMT) to escape (**c**). The resulting surviving malignant cell populations are a heterogeneous mixture of cells with the mentioned phenotypes (**d**).

Our present evidence indicates that in order to survive and successfully colonize the entire organism, malignant colonocytes face various hardships during their genesis and must express particular chemoresistance mechanisms. The timing and staging are at present not known, whereas the nature of their mutual relationship and interactions have only recently begun to emerge. Essentially, CRC cells face stress initially from the physiological constraints of the host environment and later from chemotherapy. The cells may respond to this either by inhibiting their cell death programs, by upregulating their autophagic flux, or by reprogramming themselves to EMT-competent phenotypes (Figure 2). All three mentioned options have been documented in CRC and all of them are interrelated via common signaling pathways as well as shared signals and regulators including *BECN1*, *BCL-2*, *mTOR*, AMP-activated protein kinase (*AMPK*), and select microRNAs [123,124,125,126]. As might be expected, their individual patterns and relationships are complex and our knowledge of them is at present quite cursory and based almost exclusively on experimental in vitro or in vivo evidence [123,127].

Thus, autophagy has been documented to significantly influence the ability of colon cancer cells to undergo apoptosis, whereas resistance to apoptosis was shown to have a dramatic impact on autophagy regulation in the similar models [128]. Accordingly, we have demonstrated that cytostatic irinotecan stimulated an increased autophagy rate in *TP53*-null HCT-116 cells, in which it acted against cell death execution. Upon the pharmacological suppression of autophagy in the treated cells, however, cell death rate significantly increased, a finding that corresponds to numerous other studies in various types of malignancies [129]. Furthermore, autophagy and cell death are not only opposite biological phenomena but may also channel into a specific form of stress execution signaling leading to autophagic cell death in malignant cells, a finding that may be explorable with a new class of targeted agents [86,123].

The relationship between EMT and the cell death of CRC cells has also not been entirely elucidated to date. Although it is widely acknowledged that EMT-competent CRC cells show an increased chemoresistance to several cytostatic agents, the only reported instances concern the EMT-mediated suppression of anoikis, i.e., apoptosis originating from epithelial cell detachment from a substratum [130]. Whether the very same colonocytes that express mesenchymal markers would also acquire the ability to resist classical apoptosis or other related types of cell death induced by cytostatic agents and, if so, by which mechanism(s) remains to be explored further. Some of our most recent studies, however, indicate this possibility and point at several putative targets, including for instance the TP53-dependent signaling pathway.

Finally, autophagy and EMT are considered mutually exclusive events [131], with this finding seemingly supported by the observation that EMT markers have been noted in the malignant colonocytes solely in the outer rim of CRC, i.e., in a tumor area with a low level of hypoxia and nutrient deprivation, factors that are known to be major drivers of autophagy [132]. Conversely, several published reports argue for the functional dependency and crosstalk of autophagy and EMT in tumor cells [133,134,135,136] with the proposed regulatory roles of cytoskeleton and mitochondria [127].

**Figure 3:**
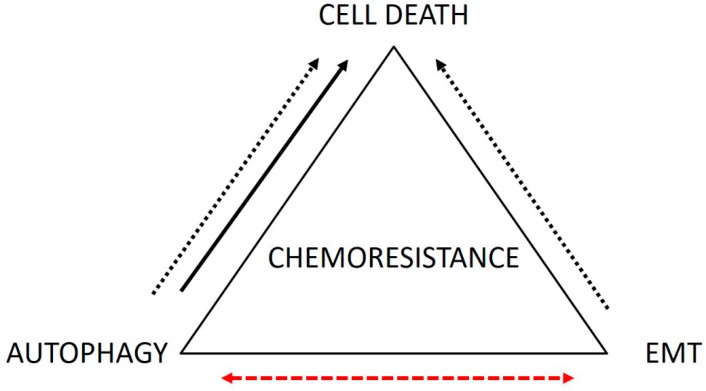
The mutual relationship between autophagy, epithelial mesenchymal transition (EMT), and apoptosis as three selected chemoresistence mechanisms in malignant colorectal carcinoma (CRC) cells. Resistant CRC cells upregulate autophagy, which acts to suppress apoptosis (dotted line). Alternatively, autophagy may contribute to apoptosis of these cells (full line). EMT in CRC cells inhibits their ability to undergo apoptosis (dotted line). Autophagy and EMT appear to be mutually exclusive events in CRC cells (dashed line).

The above-mentioned facts seem to suggest that the three discussed chemoresistance mechanisms in CRC cells represent at least initially a spectrum of choices whereby malignant cells react to individual external pressures. Due to their plasticity, these chemoresistance mechanisms and phenotypes may later be modified or even completely changed based on the concrete microenvironment context and a particular cell’s needs (Figure 3).

## 7. Conclusions

At present, it is clear that CRC cells develop chemoresistance due to the malignant process itself as well as due to their continuing struggle to adapt, which is at various (and often later) stages further enhanced by chemotherapy-related pressures. This chemoresistance is molecularly multifaceted with numerous involved players, some of which were not a subject of this work (i.e., specific classes of microRNAs). At the cellular level, chemoresistance is expressed in the ability of cells to survive via suppressed cell death and/or throughout enhanced stress combating mechanisms (autophagy) as well as stimulated reprogramming and escape (stemness and EMT). These individual biological processes share many signals and regulators and could therefore either coexist in individual malignant cells, or by mutual interconversions endow them with the desirable plasticity required for their successful survival. While the sequence of their emergence in CRC cells in vivo has not been entirely elucidated, one of the main tasks of future studies will be to verify their physical presence and regulation in cells at particular stages of their malignant conversion, as well as to reevaluate existing and newly developed therapies in this context.
